# Development of an Image Grating Sensor for Position Measurement

**DOI:** 10.3390/s19224986

**Published:** 2019-11-15

**Authors:** Shaowei Fu, Fang Cheng, Tegoeh Tjahjowidodo, Mengjun Liu

**Affiliations:** 1 Advanced Remanufacturing and Technology Centre (Agency for Science, Technology and Research), Singapore 637143, Singapore; fu_shaowei@artc.a-star.edu.sg (S.F.); liu_mengjun@artc.a-star.edu.sg (M.L.); 2 School of Mechanical and Aerospace Engineering, Nanyang Technological University, Singapore 637798, Singapore; ttegoeh@ntu.edu.sg; 3 Department of Mechanical Engineering, Katholieke Universiteit Leuven, 3001 Leuven, Belgium

**Keywords:** image grating, position measurement, subpixel image registration, measurement error correction

## Abstract

In this research paper, a precision position-measurement system based on the image grating technique is presented. The system offers a better robustness and flexibility for 1D position measurement compared to a conventional optical encoder. It is equipped with an image grating attached to a linear stage as the target feature and a line scan camera as the stationary displacement reader. By measuring the position of the specific feature in the image and applying a subpixel image registration method, the position of the linear stage can be obtained. In order to improve the computational efficiency, the calculations for pattern correlation and subpixel registration are performed in the frequency domain. An error compensation method based on a lens distortion model is investigated and implemented to improve the measurement accuracy of the proposed system. Experimental data confirms the capability of the developed image grating system as ±0.3 µm measurement accuracy within a 50 mm range and ±0.2 µm measurement accuracy within a 25 mm range. By applying different optics, the standoff distance, measurement range, and resolution can be customized to conform to different precision measurement applications.

## 1. Introduction

Precision positioning of a workpiece is a fundamental function required in many areas such as dimensional metrology and surface finish inspection [1,2], where its applications require appropriate measurement technology adoption [3]. Laser interferometers based on the heterodyne phase‑detection principle have been widely used in different industries for long displacement measurement with sub-nanometer resolution [4]. However, the integration of a laser interferometer for in situ measurement is not very straightforward due to its low robustness and high sensitivity to environmental conditions [5]. Hence, it is mainly used as a reference instrument instead of a feedback sensor for motorized stage calibration.

Optical encoders are widely used feedback sensors, which are typically paired with precision positioning systems for close-loop control. An optical encoder generally consists of an optical grating scale used as the measurement reference, an optical reading head for measuring the gradation scales, and electronics for data acquisition and processing [5]. With continuous improvement of optical scanning algorithms and signal processing technologies, optical encoders are now able to achieve submicron to nanometer accuracy. Time grating encoders are a newly developed alternative approach to optical grating encoders for displacement measurement. By accurately measuring the time and the phase of a constant-speed electric field, the displacement can be calculated [6,7].

However, most grating-based displacement sensors have some inherent disadvantages that limit their inline applications. These include (1) the short standoff distance that limits the installation flexibility, (2) sealing or packaging of the grating sensor that may be needed for contamination concerns, (3) accumulated computation that needs continuous signal sampling, where in a case of unexpected signal discontinuity, the measurement will fall short.

Digital image correlation (DIC) has been widely accepted and generally used as a highly robust technology for displacement and deformation measurement [8], and it is suitable for applications in harsh manufacturing environments [9]. By analyzing the correlation of digital images taken for the same object before and after deformation or movement, the displacements can be determined by subpixel interpolation [10]. Currently DIC is usually only used for surface deformation analysis [11].

To overcome the limitation of DIC using a 2D (two-dimensional) area scan camera, a line scan camera with a large image sensor is an ideal option for measurements of large displacements. A line scan camera creates an image as a row of single pixels at using a CMOS or CCD array detector [12]. It is particularly suitable for machine vision applications that need high speed, high spatial resolution, and simple illumination conditions for image capture [13]. In order to focus on 1D (one-dimensional) precision measurement and achieve high efficiency, a line scan camera with a large image sensor is selected instead of a conventional 2D area scan camera as the displacement reader.

To improve the line scan camera to higher accuracy, a subpixel image registration method needs to be introduced. In recent years, the most widely used technique for subpixel image registration is based on the peak location search in the cross-correlation of the two aligned images [14]. These cross-correlation-based techniques have significant registration accuracy and robustness to background lighting variations and image noise [15,16], where they can be categorized into two classes, namely spatial domain and Fourier domain methods.

The spatial domain approach typically calculates the least-squares error [17,18] or cross‑correlation [19,20] between the reference and the sensed images. The most commonly used approach for sub-pixel registration is based on an interpolation algorithm, such as bilinear interpolation, bi-cubic interpolation, and B-spline interpolation. It has been found through investigation that these methods are always limited by the sampling frequency, are sensitive to noise [15], and are not suitable to real-time applications [21]. Besides the interpolation method, the commonly used approach with no interpolation stage is based on the gradient optimization function [22]. This method iteratively searches for the maximum value in the formulated function, for example, cross-correlation, and retrieves the shifted subpixel values without interpolation for a large up-sampling factor [23]. The main shortcoming of these methods is that they rely heavily on image intensity conservation and might fail when considerable amounts of illumination differences are present between reference and sensed images [24].

When the computational speed is a concern or when the images were acquired under the frequency-dependent noise, then Fourier domain approach is preferred rather than the spatial domain approach [25,26]. A conventional approach to calculate the image translation within a fraction, 1/*p*, of a pixel is to embed the cross power spectrum in a zero-padded matrix in Fourier domain and to compute an inverse Fast Fourier Transform (FFT) to obtain the *p*-times of the up-sampled phase correlation and locate its peak [27]. This approach provides high registration accuracy and robust results, but the computational time and memory consumption are enormous [14]. To solve the problem of low computational efficiency in the conventional FFT approach, there are approaches based on interpolation algorithms, for example, iterative intensity interpolation [16] and correlation interpolation [28]. However, these methods are sometimes sensitive to image noise [24], and their accuracy largely depends on the quality of the interpolation algorithms [29].

In recent years, different new methods have been proposed to obtain fast and highly accurate image registration. Foroosh et al. [15] proposed a phase correlation method on downsampled images using a Sinc function to approximate the Dirac Delta function in the frequency domain, which has less computation complexity but limited registration accuracy (0.05 pixel resolution) compared to the conventional FFT method. Guelpa et al. and Jaramillo et al. [29,30] demonstrated different vision measurement methods to sense 1D and 2D displacements. Subpixelic performance was derived from a phase shifting algorithm [31] applied to the images of pseudo-periodic patterns. However, the measurement ranges of their developed systems are less than 200 µm. Vergara et al. [32] implemented digital holography to an artificial visual in-plane position measurement with large working distance. Their system demonstrated a resolution of 50 nm over a working distance of more than 15 cm.

Guizar-Sicairos et al. [33] proposed a single-step discrete Fourier transform (SSDFT) that uses the matrix-multiplied discrete Fourier transform (DFT) algorithm to compute the up-sampled cross-correlation between the reference and sensed images. This new method is able to achieve subpixel registration accuracy equivalent to that of the conventional FFT method with reduced computation time and memory requirements. The method has also been implemented in different machine vision applications by many researchers [26,34,35,36] and has been validated with high registration efficiency and robustness to noise.

In this research, a precision position measurement method is developed using the image grating technique that is based on the 1D SSDFT subpixel image registration algorithm. It demonstrates its capability for high robustness and flexibility compared to a conventional optical encoder that is able to achieve an inline precision position-measurement.

## 2. Experimental Setup

**Figure 1:**
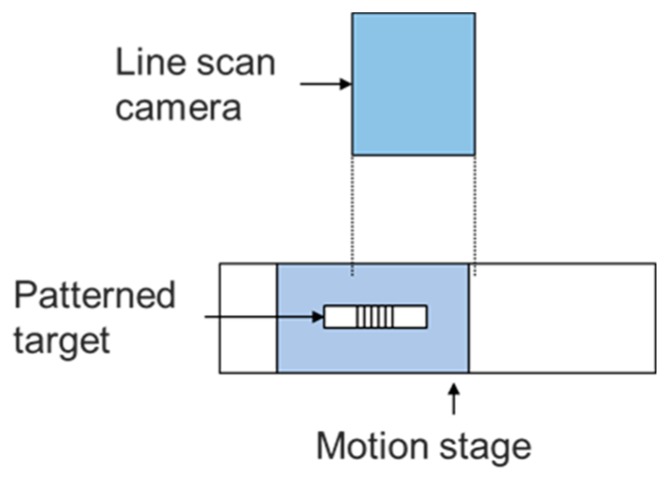
Schematic of an image grating system.

As shown in Figure 1, an image grating system consists of a patterned target attached or printed on the linear stage and a stationary line scan camera as the displacement reader. When the system is properly aligned, position of the linear stage will be correlated to the pixel translation of the patterned target on the image. By applying different optics, the standoff and measurement range can be adjusted.

**Figure 2:**
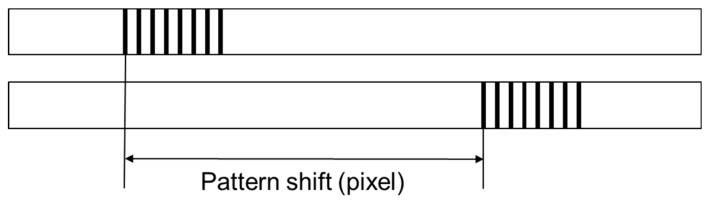
Schematic of pattern shift between two images.

This system can be pre-calibrated using a visible feature with known dimensions. In this study, line patterns with known interval distance were used. Through calibration, the pixel-to-dimension ratio can be determined. After calibration, the focusing mechanism of the optical lens shall be locked. During the measurement practice, the first image captured at the starting position is used as the reference. As shown in Figure 2, pattern shift can be calculated in pixels between the first image and subsequent images using the image registration approach. Then, the displacement can be computed according to the pixel-to-dimension ratio.

**Figure 3:**
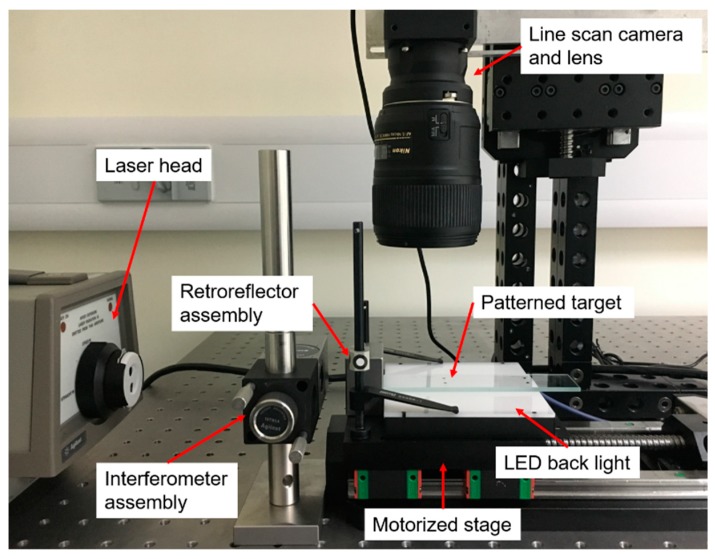
Image grating system configuration. LED: light-emitting diode.

The image grating system for measuring the motorized stage position is shown in Figure 3. A heterodyne laser interferometer 5530 (Agilent, Santa Clara, CA, USA) with linear measurement resolution of 0.5 nm was used as the reference instrument for position measurement. A linear interferometer and a linear retroreflector were assembled for linear measurement. A line scan camera (raL12288-8gm, Basler AG, Ahrensburg, Germany) with 43 mm sensor size and 12,288 pixel resolution was integrated in the system. Its sensor pixel size was 3.5 µm and sampling line rate was 8 kHz. A consumer lens (NIKKOR 60 mm F2.8G, Nikon Inc., Melville, NY, USA) was selected to provide a magnification ratio from 1:1 to 1:10.

**Figure 4:**
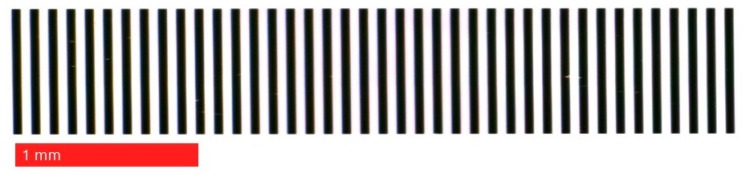
Microscope view of the patterned target.

The patterned target (Hong Cheng Optical Products, Dongguan, China) used in the developed image grating system included a set of 40 line pairs with 50 µm intervals printed on an optical glass. The manufacturing tolerance of the line feature was ±1 µm. A microscope view of the line features on the patterned target is shown in Figure 4. In order to enhance the contrast and shorten the exposure time for the line scan camera, a light-emitting diode (LED) illumination backlight (CV-NFL-100X96W, Moritex Corporation, Asaka, Japan) was placed as the substrate of the patterned target. A motorized stage (TSA100-B, Zolix Instruments Co., Ltd., Beijing, China) was selected for position measurement. It had a travel range of 100 mm, a minimum incremental motion of 1.25 µm, and a positioning repeatability less than 5 µm.

In order to implement the image grating system into practical position measurement, the measurement accuracy needs to be determined. Most commercial surface topography measuring instruments are integrated with motorized stages configured with a stepper or direct current (DC) motor with lead screw. The positioning accuracy of these types of cost-effective motorized stages range from 0.5 to 5 µm [37,38,39]. Therefore, the goal of the developed image grating system was to achieve position measurement accuracy better than 0.2 µm. However, the image sensor in a typical line scan camera has a pixel size in the micrometre level. To break through the pixel-level resolution of the line scan camera, a subpixel image registration algorithm was introduced in the image grating system.

## 3. Subpixel Image Registration Based on 1D Single-Step Discrete Fourier Transform

The image registration methods in the Fourier domain are based on the well-known Fourier shift theorem. Given two images *f*(*x,y*) and *g*(*x,y*), assume that horizontal shift *x*_0_ and vertical shift *y*_0_ between the two images, *f*(*x,y*) and *g*(*x,y*), are related by the following transformation [33]:

(1) $$f\left(x,y\right)=g\left(x-x_{0},y-y_{0}\right).$$

According to the Fourier shift theorem, the discrete Fourier transform (DFT) of *f*(*x,y*) and *g*(*x,y*) satisfy:

(2) $$F\left(u,v\right)=G\left(u,v\right)\exp\left[-i2\pi \left(\frac{ux_{0}}{M}+\frac{vy_{0}}{N}\right)\right],$$

where *F*(*u,v*) and *G*(*u,v*) are the DFT of *f*(*x,y*) and *g*(*x,y*), respectively, and *M* and *N* stand for the image dimensions. The normalized cross-correlation spectrum is defined as follows:

(3) $$R\left(u,v\right)=\frac{F(u,v)G^{*}\left(u,v\right)}{\left|F(u,v)G^{*}(u,v)\right|}=\exp\left[-i2\pi \left(\frac{ux_{0}}{M}+\frac{vy_{0}}{N}\right)\right],$$

where * indicates the complex conjugation. The inverse Fourier transform (IFT) of the normalized cross-correlation spectrum *R*(*u,v*) is a Dirac Delta function [15] centered at (*x*_0_,*y*_0_) as follows:

(4) $$r\left(x,y\right)=IFT\;\left[R(u,v)\right]=\delta \left(x-x_{0},y-y_{0}\right).$$

It can be observed that the accuracy of the Fourier domain method fully depends on the peak in the Dirac Delta function as in *r*(*x,y*). The basic phase correlation method can only be used for pixel‑level registration as a coarse estimation. To improve the coarsely registered images to higher accuracy, the subpixel image registration method based on SSDFT was introduced.

Here, we provide a brief background on 2D SSDFT. The SSDFT approach comprises two steps. The first step is to coarsely estimate the peak location of the phase correlation between two images using the conventional zero-padding FFT approach with an up-sampling factor of *p* = 2. In the second step, the SSDFT approach refines the estimation for the accurate peak in a 1.5 × 1.5 pixel neighborhood around the coarse estimation, based on matrix-multiply DFT.

In our application for position measurement using a line scan camera that creates a 1D image as a row of single pixels, the 2D SSDFT algorithm was simplified to the 1D SSDFT algorithm as follows. A 1D image can be defined as a 1D discrete signal *f*(*X*) and the Fourier transform (FT) of the signal in matrix form can be written as:

(5) F(U)=E(UX)⋅f(X),

where $X={\left(x_{0},\ldots ,x_{N_{A}-1}\right)}^{T}$, $U={\left(u_{0},\ldots ,u_{N_{B}-1}\right)}^{T}$ and

(6) $$E(UX)=\left(\begin{array}{ccccc} e^{-i2\pi x_{0}u_{0}} & \ldots & e^{-i2\pi x_{k}u_{0}} & \ldots & e^{-i2\pi x_{N_{A}-1}u_{0}}\\ \ldots & \ldots & \ldots & \ldots & \ldots \\ e^{-i2\pi x_{0}u_{k}} & \ldots & e^{-i2\pi x_{k}u_{k}} & \ldots & e^{-i2\pi x_{N_{A}-1}u_{k}}\\ \ldots & \ldots & \ldots & \ldots & \ldots \\ e^{-i2\pi x_{0}u_{N_{B}-1}} & \ldots & e^{-i2\pi x_{k}u_{N_{B}-1}} & \ldots & e^{-i2\pi x_{N_{A}-1}u_{N_{B}-1}} \end{array}\right).$$

Equation (5) is the FT in matrix form deduced from the representation by the Riemann sum of continuous FT, and if *N_A_* is equal to *N_B_*, it becomes the DFT of the signal [40]. Based on the matrix FT described in Equations (5) and (6), the up-sampling matrix IFT can be defined similarly. Firstly, the initial normalized cross-correlation *R*(*u,v*) from Equation (3) is modified as *R*(*u*):

(7) $$R(u)=\frac{F\left(u\right)G^{*}\left(u\right)}{\left|F\left(u\right)G^{*}\left(u\right)\right|}=\exp\left[-i2\pi \left(\frac{ux_{0}}{M}\right)\right].$$

Defining *m* as the pixel size of the up-sampled region around the coarse estimation (*m* = 1.5 in the SSDFT algorithm), *p* as the up-sampling factor in the spatial domain, and *Ŝ* as the coarse estimation of the peak location of *R*(*u*), the up-sampling range *L* is represented as:

(8) $$L=\left\{x^{\prime },p\cdot \hat{S}-\frac{pm}{2}\leq x^{\prime }\leq p\cdot \hat{S}+\frac{pm}{2}\right\}.$$

It is known that the discrete spectrum in the Fourier domain corresponds to a continuous periodic signal in the spatial domain. Therefore, increasing the sampling rate of the signal will result in a higher resolution of the spatial domain signal. Given a normalized cross power spectrum, *R*(*U*), the up-sampling matrix inverse FT on *L* is defined based on the matrix-multiply FT:

(9) $$r(X^{\prime })=\left\{\begin{array}{r} e^{i2\pi X^{\prime }U^{\prime }}\cdot R(U),\;X^{\prime }\in L\\ \;0,\;X^{\prime }\notin L \end{array}\right\},$$

where *X*′ is the spatial distribution of the up-sampled phase correlation *r*(*X*′).

It is assumed that the sampling step of *x*′ is 1/*p* pixel. The up-sampling matrix inverse FT operator restricts the region of the *R*(*U*), and [−*m*/2, *m*/2] is the region of the coarse estimation and the accurate phase correlation peak is located within this region. Firstly, after the coarse estimation *Ŝ* is obtained in the conventional zero-padding FFT approach and the up-sampling region is set according to *m*, the region [*Ŝ* − *m*/2, *Ŝ* + *m*/2] is up-sampled with factor *p* to determine vector *X*′. Finally, the up-sampled phase correlation *r*(*X*′) can be calculated based on Equation (9). By searching for the maximum in vector *X*′, the fine estimation ∆*Ŝ* can be obtained. Then, the final estimation of the translation *S* between the two images is achieved:

(10) $$S=\hat{S}+\Delta S$$

**Figure 5:**
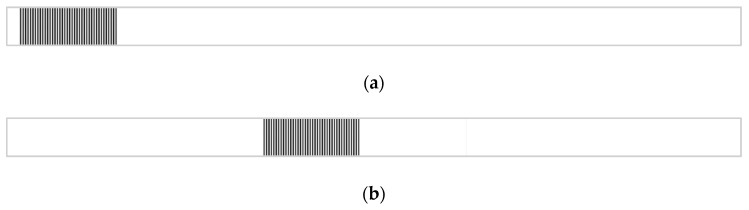
One-dimensional images for phase correlation calculation. (**a**) Reference image; (**b**) Shifted image.

**Figure 6:**
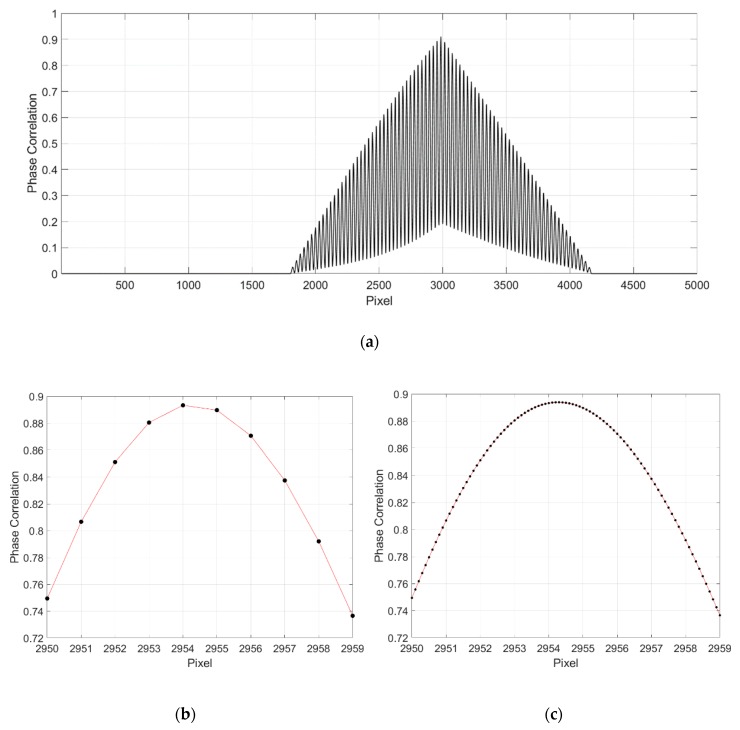
Phase correlation of two 1D images. (**a**) Full phase correlation plot; (**b**) Enlarged phase correlation plot; (**c**) Up-sampled phase correlation (up-sampling factor *p* = 10).

Figure 5 shows an example of two 1D images for translation calculation. The normalized cross‑power spectrum between the reference image and shifted image can be obtained by Equation (3), which is illustrated in Figure 6a, while the enlarged view around the peak location is shown in Figure 6b. It can be seen that the peak location on the spectrum is within pixel-level accuracy. Then, the normalized cross-correlation spectrum is up-sampled by the SSDFT method in the neighborhood around the peak location in Figure 6b, and the up-sampled cross correlation spectrum is depicted as in Figure 6c. Comparing Figure 6b,c, it can be concluded that the location of the peak is more accurate after up-sampling using the SSDFT algorithm.

To validate the image registration accuracy and computational cost of the proposed 1D SSDFT method, a comparison study was carried out between the proposed 1D SSDFT method and the 2D SSDFT method.

To test the 2D SSDFT method, eight line images were captured sequentially to form an 8 × 9000 pixel 2D image. The 1D SSDFT method takes the average of each column in the 8 × 9000 pixel 2D image to give a 1 × 9000 pixel 1D image. Each image registration was repeated 100 times in the MATLAB software platform (MathWorks, Natick, MA, USA) to reduce the computational fluctuation of the CPU and memory. The image translation and computational time of image registration using 1D and 2D SSDFT methods are shown in Table 1. It was revealed that the 1D SSDFT method offers comparable registration accuracy, but requires less computational time compared to the 2D SSDFT method.

## 4. Error Correction Based on Lens Distortion

The image registration algorithms discussed in the previous section are all based on an assumption that the lens magnification is uniform over the full field of view, which is unrealistic for actual optics. Most lenses have either barrel or pincushion distortion [41,42]. This section provides a discussion of measurement errors caused by lens distortion.

### 4.1. Theoretical Modelling

With an ideal optical lens, where the magnification is uniform over the entire image field, the displacement of the target in the object field *D*_0_ is proportional to that in the image field *D_i_*:

(11) $$D_{0}=kD_{i},$$

where *k* is the reciprocal of the lens magnification.

If the lens has either barrel or pincushion distortion, leading to a variable magnification, the relationship between *D*_0_ and *D_i_* can be expressed as:

(12) $$D_{0}=\int_{x_{0}}^{x_{t}}k(x)dD_{i},$$

where *k*(*x*) is the reciprocal of the lens magnification as a function of *x*. With Equations (11) and (12), the measurement error can be represented as:

(13) $$E=\int_{x_{0}}^{x_{t}}k(x)dD_{i}-\bar{k}\cdot (x_{t}-x_{0}),$$

where $\bar{k}$ is the undistorted reciprocal of the lens magnification and *x*_0_ and *x_t_* are the starting and stopping locations of the patterned target in the image, respectively. In a digital image, where the positioning information is discretized by pixilation, Equation (13) can be replaced by:

(14) $$E=\sum_{i=m}^{n}k(i)\cdot d-\bar{k}\cdot (n-m)d,$$

where *k*(*i*) is the reciprocal of magnification at the *i*th pixel and *d* is the dimension of one pixel, while *m* and *n* are the pixel indexes of the starting and stopping locations of the target in the image, respectively. The unevenness magnification of the lens can be fitted with a 4th-degree polynomial equation [17,41,43] and written as:

(15) $$k(i)=a+bx^{2}(i)+cx^{4}(i),$$

where *x* = 0 represents the center of the camera and $k\left(0\right)=\bar{k},$ which is the undistorted reciprocal of magnification.

**Figure 7:**
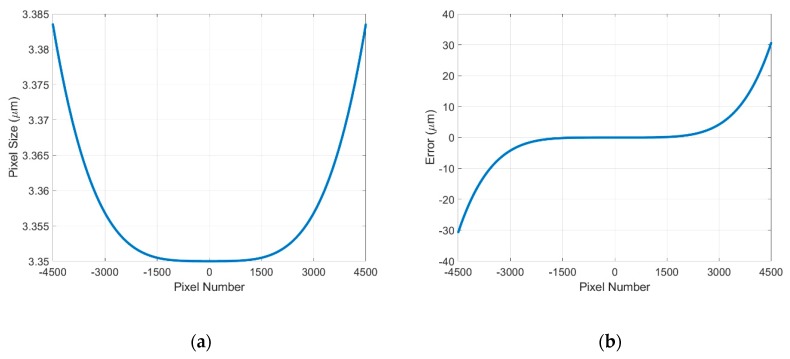
(**a**) Lens distortion curve; (**b**) Position error curve.

To simulate the lens distortion in a consumer lens (NIKKOR 60 mm F2.8G) integrated in the developed image grating system, we assumed the image starting position *m* = −4500 and stopping position *n* = 4500, the pixel size *d* = 3.35 µm, and the reciprocal of magnification $\bar{k}=k\left(0\right)=1$, k(2500)=1.001, k(4500)=1.001. Substituting *m*, *n*, *d*, $\bar{k}$, *k*(0), *k*(2500), and *k*(4500) into Equations (14) and (15), the simulated lens distortion curve and position error curve can be plotted as Figure 7. It is obvious that the position error curve can be fitted with a 5th-degree polynomial equation, which is consistent with the integral of the 4th-degree polynomial equation *k*(*x*) in Equation (13).

### 4.2. Experimental Verification

**Figure 8:**
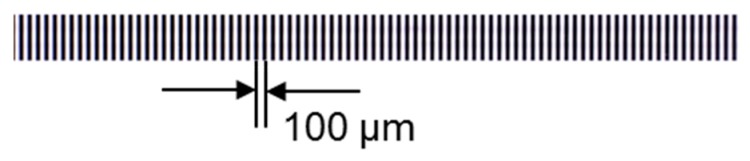
Enlarged details of the 100 mm pattern target.

To verify the theoretical modelling of the lens distortion at its closest focus distance (magnification 1:1), a 100 mm patterned target with line pairs of 100 µm intervals was used to quantify the lens distortion. The enlarged details of the 100 mm patterned target are shown in Figure 8. To avoid vignetting, only the central section of the whole field of view in the line scan camera was used for lens distortion verification.

**Figure 9:**
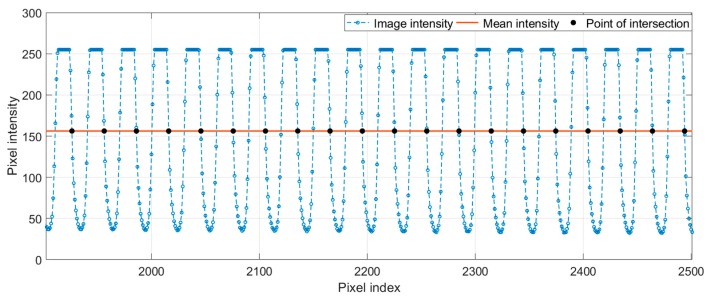
Points of intersection between the mean intensity of the whole 1D image and the 1D image intensity curve.

Due to the lens distortion, the pixel numbers in each line-pair (Figure 8) are not identical. By counting the pixel numbers in each line-pair, the lens distortion and the position error of the pattern shift can be analyzed. In this experimental verification, a linear interpolation method was used to locate the points of intersection between the mean grayscale intensity of the whole 1D image and the 1D image intensity curve (Figure 9).

**Figure 10:**
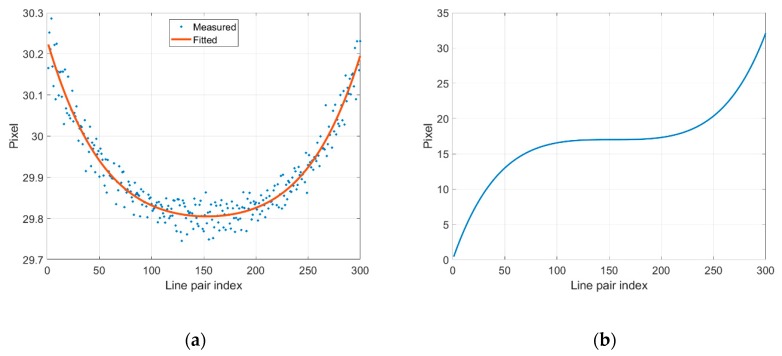
(**a**) Pixel numbers between a line pair; (**b**) Cumulated pixel error between line pairs.

Subsequently, the pixel numbers within one line-pair can be calculated at the sub-pixel level, as shown in Figure 10a. To reduce the measurement and interpolation error, a 4th-degree polynomial equation was used to fit the measured data points. It can be seen that the pixel number within one line-pair at the image center (line-pair index = 150) is less than the pixel number at the image edges (line-pair index = 0, 300). It can also be concluded that the consumer lens (NIKKOR 60 mm F2.8G) has a pincushion distortion and that the image magnification increases with distance from the image center. By integrating the 4th-degree polynomial equation and then subtracting the integration of the pixel number within one line-pair at the image center, the error curve of cumulated pixels between line pairs is plotted in Figure 10b.

## 5. Experimental Results

### 5.1. Measurement Results

**Figure 11:**
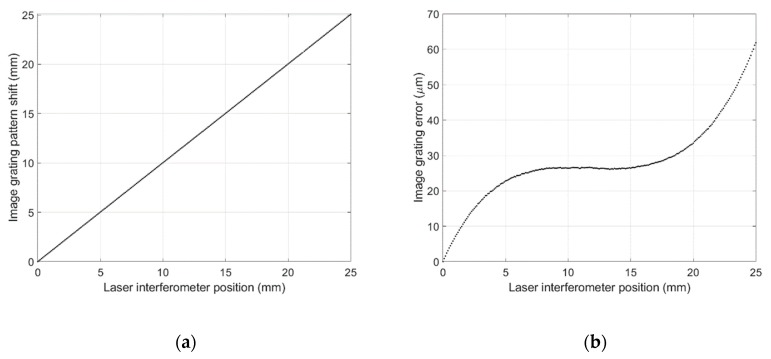
(**a**) Pattern shift (mm) in the image grating system; (**b**) Measurement error of the image grating system.

In the 25 mm travel length of the motorized stage, measurement results for both the reference instrument (laser interferometer 5530) and the image grating system were recorded with 0.1 mm intervals as shown in Figure 11a, where the deviation in the measurement values is depicted in Figure 11b. The trend of measurement error from experimental tests is consistent with the theoretical analysis presented in Section 4.1. From both theoretical analysis and experimental tests, it can be concluded that the measurement error is pixel-location-dependent. When the optical lens is fixed on the camera, a preloaded error curve can be used for error compensation.

### 5.2. Fifth-Degree Polynomial Fitting

**Figure 12:**
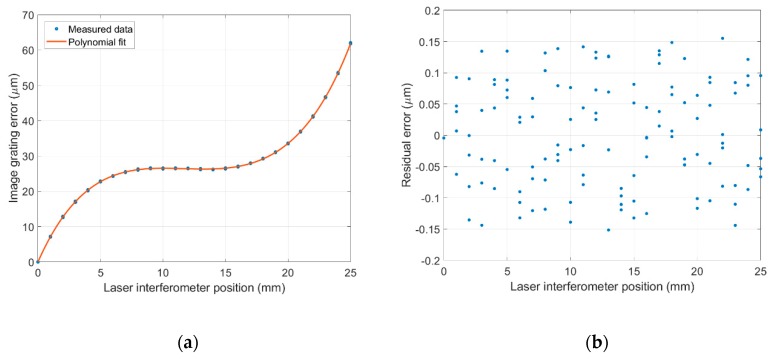
(**a**) Fifth-degree polynomial fitting; (**b**) Residual errors after polynomial fitting.

As discussed in Section 4.1, the error curve shown in Figure 11b can be fitted to a 5th-degree polynomial equation using a least squares algorithm. Five repeated measurements were conducted with 1 mm steps in a 25 mm travel range, where five sets of 26 data points and a 5th-degree polynomial curve fitting the data points were generated and plotted in Figure 12a. The residual errors after 5th-degree polynomial fitting are shown in Figure 12b and are in the range of ±0.15 µm.

### 5.3. Position Measurement and Error Compensation

**Figure 13:**
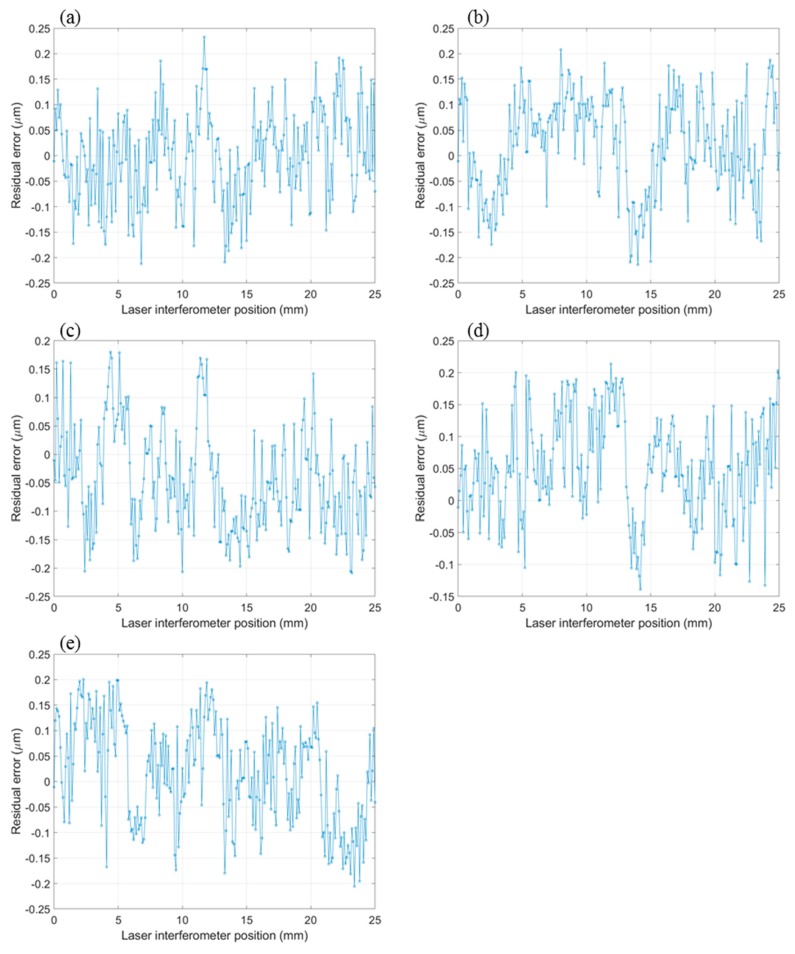
(**a**–**e**) Residual errors of repeated five measurements (25 mm).

The position measurement of the motorized stage was carried out with 0.1 mm incremental steps in a 25 mm travel range and the moving speed was set to 1 mm/s. In each measurement, eight line images were captured sequentially and averaged to a 1D image to reduce the background noises and illumination inconsistency. The measurements were repeated five times, and the 5th-degree polynomial fitting curve plotted in Figure 12a was used to compensate for the lens distortion and misalignment error of the image grating system. The five set measurement error curves are shown in Figure 13. It can be concluded that after correcting for the lens distortion and misalignment, most of the measurement residual errors from the developed image grating system can be controlled within ±0.2 µm.

**Figure 14:**
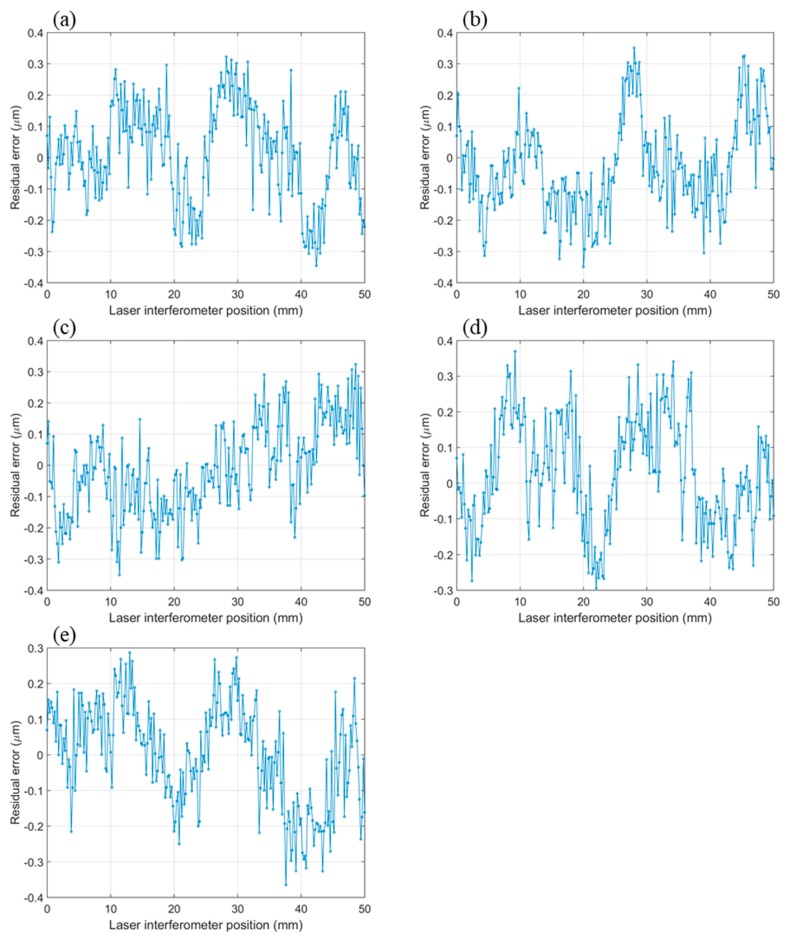
(**a**–**e**) Residual errors of repeated five measurement (50 mm).

In order to test the versatility of the developed image grating system, the camera lens magnification was changed from 1:1 to 1:2 to measure a longer travel distance. The reconfigured image grating system was used to measure 50 mm of displacement of the motorized stage with 0.2 mm incremental steps. The five set measurement error curves are plotted in Figure 14. It can be observed that the measurement residual error is in the range of ±0.3 µm after 5th-degree polynomial fitting.

### 5.4. Measurement Repeatability Study

**Figure 15:**
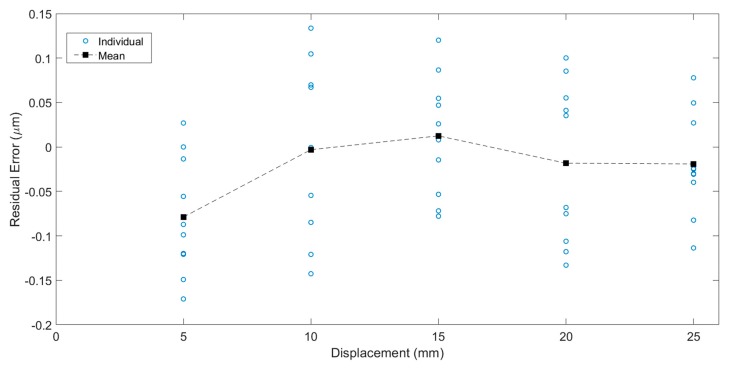
Positioning repeatability of image grating system.

To test the measurement repeatability of the image grating system, the entire system was reassembled, and tests on five locations (5 mm, 10 mm, 15 mm, 20 mm, and 25 mm) were taken for the comparison study. The laser interferometer 5530 was used as a benchmark. For each location, the position measurement was repeated 10 times. The measurement error curve plotted in Figure 15 shows a good repeatability after error compensation using the 5th-degree polynomial equation, and the residual errors are within ±0.15 µm.

## 6. Conclusions

In this study, the measurement results have demonstrated that the developed image grating system has a measurement error of ±0.2 µm within a 25 mm measurement range. When the optical lens is set for 1:1 magnification, the standoff distance is around 100 mm. If optics with longer focal lengths are applied, maintaining the same magnification, the standoff distance can be increased. This might be helpful in applications where long clearance is needed. When the camera is moved further from the object, the field of view becomes enlarged. When the optical lens is set to 1:2 magnification, the measurement error of ±0.3 µm within a 50 mm measurement range is still acceptable.

Conventional position measurement technologies, for example, optical grating and laser interferometry, rely on continuous signal sampling. Signal discontinuity, due to sensor contamination or optical path blocking, will lead to measurement failure. The proposed image grating system is able to determine the absolute position of the targeted object whenever the patterned feature is detectable. This advantage helps to improve the system robustness for in situ position measurement.

The measurement range of the developed system is limited by the field of view of the line camera. However, using a longer patterned target will require high precision graduations of constant pitch (e.g., a linear scale used in an incremental optical encoder). Moreover, to achieve absolute position measurement, an absolute encoded scale of higher complexity and expense is needed. In future work, to achieve a larger range with absolute position information, image stitching will be applied for simple but unique features printed on the scale.

## Figures and Tables

**Table 1:** Registration speed and accuracy comparison between 1D and 2D single-step discrete Fourier transform (SSDFT) methods.

SSDFT Method	Image Translation (pixel)	Computational Time (s)
1D	2984.180	2.9693
2D	2984.180	4.9092
